# No observed effect of a student-led mock objective structured clinical examination on subsequent performance scores in medical students in Canada

**DOI:** 10.3352/jeehp.2019.16.14

**Published:** 2019-05-27

**Authors:** Lorenzo Madrazo, Claire Bo Lee, Meghan McConnell, Karima Khamisa, Debra Pugh

**Affiliations:** 1 Faculty of Medicine, University of Ottawa, Ottawa, ON, Canada; 2 Department of Medicine, McGill University, Montreal, QC, Canada; 3 Department of Anesthesiology and Pain Medicine, University of Ottawa, Ottawa, ON, Canada; 4 Department of Medicine, University of Ottawa, Ottawa, ON, Canada; 5 Medical Council of Canada, Ottawa, ON, Canada; Hallym University, Korea

**Keywords:** Physical examination, Educational measurement, Medical education, Medical students, Canada

## Abstract

Student-led peer-assisted mock objective structured clinical examinations (MOSCEs) have been used in various settings to help students prepare for subsequent higher-stakes, faculty-run OSCEs. MOSCE participants generally valued feedback from peers and reported benefits to learning. Our study investigated whether participation in a peer-assisted MOSCE affected subsequent OSCE performance. To determine whether mean OSCE scores differed depending on whether medical students participated in the MOSCE, we conducted a between-subjects analysis of variance, with cohort (2016 vs. 2017) and MOSCE participation (MOSCE vs. no MOSCE) as independent variables and the mean OSCE score as the dependent variable. Participation in the MOSCE had no influence on mean OSCE scores (P=0.19). There was a significant correlation between mean MOSCE scores and mean OSCE scores (Pearson r=0.52, P<0.001). Although previous studies described self-reported benefits from participation in student-led MOSCEs, it was not associated with objective benefits in this study.

The objective structured clinical examination (OSCE) is a widely used form of both formative and summative assessment in Canadian undergraduate medical education. As with written assessments, there is some evidence that performance-based assessments also drive learning [1]. Participants in formative OSCEs have reported the value of practice opportunities to inform their study/practice strategies, improve their content-based knowledge, and refine their overall test-taking skills [2]. Peer assessment is an emerging modality that has been used in formative OSCEs to substitute or supplement faculty examiners in low-stakes settings, while enhancing student learning using the assessment for learning framework [3]. For brevity, we will refer to these peer-assisted formative OSCEs as mock OSCEs (MOSCEs). Students participating in a MOSCE at our institution reported benefits to learning and improvements in confidence, and stated that they valued feedback from their peers [4], corroborating findings in the broader literature [3].

As MOSCEs provide students a low-stakes opportunity to experience repeated testing, they should, in theory, lead to improved subsequent performance on subsequent higher-stakes OSCEs. However, whether MOSCEs are associated with objectively improved faculty-led OSCE outcomes is currently unknown. Our study sought to investigate whether participation as an examinee in a MOSCE was associated with improved performance on subsequent OSCEs.

Ethics statement: This study was approved by the Ottawa Health Science Network–Research Ethics Board (OHSN-REB Protocol #20170595). Informed consent was obtained from participating students.

The University of Ottawa administers a variety of OSCEs throughout the 4 years of the undergraduate medical program. During the first 2 years, the faculty organizes formative and summative 10-station OSCEs that assess students on focused history taking and physical examination skills. The third-year OSCEs introduce clerkship students to management and counseling stations, which are markedly more complex station types than those experienced in pre-clerkship. Trained faculty members and resident physicians serve as examiners and trained standardized patients (SPs) are present to help OSCE participants complete the various stations. Participants are scored using a combination of checklists and rating scales. Further details have been published elsewhere [5].

We initiated a yearly student-led MOSCE in 2015 to provide third-year (M3) students an opportunity to experience a clerkship-level OSCE. The MOSCE was held less than a month before the M3 OSCE. In our MOSCE, first- and second-year medical students (M1 and M2) acted as SPs, fourth-year (M4) students served as examiners, and M3 students were the examinees; all students participated on a voluntary basis. The MOSCE consisted of 5 stations, which assessed skills in history-taking, physical examination, counseling, and management. The cases were based on a broad sample of the specialties tested on the Medical Council of Canada Qualifying Examination part II [6]. Mirroring the format of our faculty-run OSCEs, 10 minutes were allotted to complete each station (1 minute to read the prompt, 7 minutes to complete the station, and 2 minutes to receive feedback from the examiner). M4 examiners used both a checklist and a global rating scale (GRS) to assess examinees.

The MOSCE cases and checklists were designed by medical students (LM & CBL) and reviewed by a faculty advisor (KK). Complete details about the MOSCE design have been reported elsewhere [4]. All study participants were M3 students from the University of Ottawa Faculty of Medicine during the 2015–2016 and 2016–2017 academic years. A research assistant collected and de-identified the mean total OSCE scores of all M3 students from the 2015–2016 and 2016–2017 academic years (year 1 and year 2 of MOSCE implementation, respectively). The mean total MOSCE scores of M3 students who participated in the aforementioned cohorts were also collected and de-identified. These scores were subsequently linked using a unique study ID.

The primary dependent measure was the total score on the third-year OSCE. A combination of checklists and GRS scores were used to assess performance on faculty-run OSCEs. The total score for each student was determined by combining the checklist and GRS scores for each station. The relative weights of each component were determined *a priori*. Station scores were equally weighted to arrive at an overall score out of 100.

To determine whether mean OSCE scores differed depending on whether medical students participated in the MOSCE, we conducted a between-subjects analysis of variance, with cohort (2016 versus 2017) and MOSCE participation (MOSCE versus no MOSCE) as independent variables and mean OSCE score as the dependent variable.

To examine the relationship between performance on the MOSCE and on the OSCE, we conducted an analysis of a subset of the data that included students who took both the MOSCE and the OSCE. Pearson correlation analyses were then conducted between the mean MOSCE checklist scores and the OSCE scores. All statistical analyses were conducted using IBM SPSS ver. 24.0 (IBM Corp., Armonk, NY, USA).

We analyzed data from 330 M3 students: 172 from the 2016 cohort and 158 from the 2017 cohort. The raw data are available in Supplement 1. Similar proportions of students participated in the MOSCE across the 2 cohorts, with 46 (26.7%) from the 2016 cohort and 43 (27.2%) from the 2017 cohort. The MOSCE examinees (2016 and 2017) did not differ significantly from the non-participants with regards to their prior performance when comparing their baseline second-year OSCE scores through the independent-samples t-test (Table 1). The statistical results are presented in detail in Appendix 1.

As illustrated in Fig. 1, participation in the MOSCE had no influence on the mean OSCE score (P=0.19). There was also no difference in mean OSCE scores as a function of student cohort (P=0.60), nor was there an interaction between cohort and MOSCE group (P=0.57).

Given the lack of a cohort effect in the aforementioned analyses, we combined the data from both the 2016 and 2017 cohorts for the following analyses. There was a significant correlation between mean MOSCE scores and mean OSCE scores (Pearson r=0.52, P<0.001), suggesting that students who received high scores on the MOSCE were more likely to receive higher scores on the OSCE.

Our findings show that student participation as an examinee in our peer-assisted MOSCE did not affect future OSCE scores. Several factors may have contributed to this. First, the MOSCE was administered 1 month prior to the subsequent OSCE for both third-year examinees and second-year SPs. Thus, the gap between rehearsal/testing and recall may have blunted any benefits to a subsequent OSCE, as there is previous research noting that OSCE participants have poor recollection of verbal feedback received immediately following a station [7]. While we promptly returned the scoresheets with written feedback to examinees following the MOSCE, how this may have impacted recall is unclear. Second, the main purpose of our MOSCE was to prepare M3 students for the experience of more complex station types (such as counseling and management), and it was not designed to be an all-inclusive review of testable content-related knowledge. If OSCE stations were completely different from MOSCE stations, there may have been no ‘priming’ effect that would have helped students achieve higher content-based checklist scores on the OSCE, even though there may have been some improvement in test-taking skills. Recent research on a formative OSCE found that one difference between high- and low-performing students was how they gauged the importance of content-related knowledge [2]. Thus, perhaps rehearsing content-related material may be more beneficial for subsequent OSCE performance compared to rehearsing specific station types. Finally, while we initially hypothesized that the participants volunteering for the MOSCE could be a self-selected group of higher-performing students at baseline, our analyses revealed that baseline OSCE performance was similar between both groups of M3 students, suggesting that both high- and low- performing students showed a similar level of interest in the MOSCE as a preparation activity.

Our previous report demonstrated that this particular MOSCE was cost-effective and was positively viewed by all participants, and that examinees and SPs perceived self-reported learning benefits [4]. Thus, while participation in the MOSCE does not seem to affect future OSCE scores, it may have utility in helping students feel more confident and less anxious about higher-stakes OSCEs.

Our study has several limitations. First, as the stations in this MOSCE were designed by students, the construct of this MOSCE may be called into question. However, the strong correlation of student performance between the MOSCE and OSCE(s) supports the external validity of our construct. Second, the relatively small sample of students who participated in the MOSCE limited the extent of analyses performed. Finally, our MOSCE was performed at a single center with a specific cohort of students, limiting the generalizability of our data. Future studies with larger sample sizes are needed to corroborate our findings and to further explore how MOSCE participation affects factors such as organization, communication, and professionalism.

## Figures and Tables

**Fig. 1.**
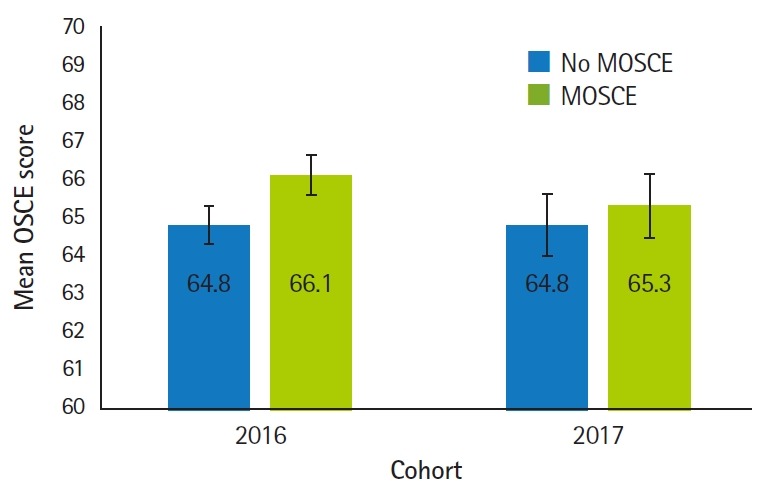
Comparison of mean OSCE scores between MOSCE participants and non-MOSCE participants. OSCE, objective structural clinical examination; MOSCE, mock OSCE.

**Table 1.** Comparison of baseline second-year OSCE scores between MOSCE participants and non-participants

Variable	Mean M2 OSCE score±SD	t-value (df)	P-value
2016 M3 MOSCE		1.500 (161)	0.136
Participants (n=43)	78.5±3.7^a)^		
Non-participants (n=120)	77.4±4.1^a)^		
2017 M3 MOSCE		1.174 (161)	0.242
Participants (n=45)	75.4±4.8^b)^		
Non-participants (n=118)	74.5±4.1^b)^		

The independent-samples t-test was used, and 2-tailed P-values were calculated. OSCE, objective structural clinical examination; MOSCE, mock OSCE; SD, standard deviation; df, degrees of freedom.

a) M2 OSCE score 2015.

b) M2 OSCE score 2016.

## Supplementary material

Supplement 1. Data files are available from https://doi.org/10.7910/DVN/YCOHO7

Supplement 2. Audio recording of the abstract.

## Appendix 1. Detailed statistical results

**Appendix Table 1.** Comparison of mock OSCE participants and non-participants in terms of prior performance on an OSCE

Variable	Mean second-year OSCE score±SD	t-value (df)	P-value
2016 Third-year MOSCE		1.500 (161)	0.136
Participants (n=43)	78.5±3.7^a)^		
Non-participants (n=120)	77.4±4.1^a)^		
2017 Third-year MOSCE		1.174 (161)	0.242
Participants (n=45)	75.4±4.8^b)^		
Non-participants (n=118)	74.5±4.1^b)^		

The independent-samples t-test was used, and 2-tailed P-values were calculated. For the 2016 third-year OSCE (n=172), the median was 65.5 (mean=65.1) and for the 2017 third-year OSCE (n=160), the median was 64.8 (mean=64.9). Using a median split, students were grouped as either high- or low-performing. The chi-square test for independence was performed and no relationship was found between performance (high or low) on the OSCE and participation in the MOSCE as either an examinee or standardized patient (see Tables 2–4). OSCE, objective structural clinical examination; MOSCE, mock OSCE; SD, standard deviation; df, degrees of freedom.

a) Second-year OSCE score 2015.

b) Second-year OSCE score 2016.

**Appendix Table 2.** The relationship between mock OSCE participation (as an examinee) and performance (low or high) on a subsequent OSCE (chi-square test for independence)

Participation as mock OSCE 2016 examinee	Third-year OSCE 2016		Total
	Low performers	High performers	
Yes	20	26	46
No	58	57	115
Total	78	83	161

χ2 (1, N=161)=0.637, P=0.425. OSCE, objective structured clinical examination.

**Appendix Table 3.** The relationship between mock OSCE participation (as an examinee) and performance (low or high) on a subsequent OSCE (chi-square test for independence)

Participation as mock OSCE 2017 examinee	Third-year OSCE 2017		Total
	Low performers	High performers	
Yes	22	22	44
No	54	57	111
Total	76	79	155

χ2= (1, N=155), P=0.879. OSCE, objective structured clinical examination.

**Appendix Table 4.** The relationship between mock OSCE participation (as a SP) and performance (low or high) on a subsequent OSCE (chi-square test for independence)

Participation in mock OSCE 2017 as SP	Second-year OSCE 2017		Total
	Low performers	High performers	
Yes	10	5	15
No	66	80	146
Total	76	85	161

χ2 = (1, N=161), P=0.113. The correlation between 2016 mock OSCE scores and subsequent scores on a third-year OSCE were high (r=0.490, P=0.001). Similarly, the 2017 mock OSCE and subsequent third-year OSCE scores showed a high correlation (r=0.619, P<0.001). However, the correlation between the second-year and third-year OSCEs was also high (r=0.560, P<0.001; r=0.510, P<0.001). OSCE, objective structured clinical examination; SP, standardized patient.
